# ATP Bioluminescence Feedback Is Associated with Reduced Residual Hand Contamination Among Healthcare Workers: A Single-Center Before-and-After Study

**DOI:** 10.3390/healthcare14111493

**Published:** 2026-05-28

**Authors:** Emilia Doaga Pruna, Norberth-Istvan Varga, Madalina-Ianca Suba, Florina Buleu, Ioan Demeter, Maria Daniela Mot, Lavinia Davidescu, Theodor-Florin Pantilimonescu, Florin George Horhat

**Affiliations:** 1 Doctoral School, “Victor Babes” University of Medicine and Pharmacy, Eftimie Murgu Square, No. 2, 300041 Timisoara, Romania; emilia.pruna@umft.ro; 2 Department of Nursing, “Victor Babes” University of Medicine and Pharmacy, Eftimie Murgu Square, No. 2, 300041 Timisoara, Romania; norberth.varga@umft.ro (N.-I.V.); demeter.ioan@gmail.com (I.D.); 3 Department XIII, Discipline of Infectious Diseases, “Victor Babeş” University of Medicine and Pharmacy, Eftimie Murgu Square, No. 2, 300041 Timisoara, Romania; madalina.suba@umft.ro; 4 Department VI, Discipline of Internal Medicine and Ambulatory Care, Prevention and Cardiovascular Recovery, Faculty of Medicine, “Victor Babes” University of Medicine and Pharmacy, Eftimie Murgu Square, No. 2, 300041 Timisoara, Romania; florina.buleu@umft.ro; 5 Department of General Medicine, “Vasile Goldis” Western University of Arad, B-dul Revolutiei, No. 96, 310025 Arad, Romania; mot.dana@uvvg.ro; 6 Department of Medical Disciplines, Faculty of Medicine and Pharmacy, University of Oradea, 410073 Oradea, Romania; lavinia.davidescu@didactic.uoradea.ro; 7 Department of Physiology, “Grigore T. Popa” University of Medicine and Pharmacy, 700115 Iasi, Romania; 8 Department of Microbiolgy, “Victor Babes” University of Medicine and Pharmacy, Eftimie Murgu Square, No. 2, 300041 Timisoara, Romania; horhat.florin@umft.ro; 9 Multidisciplinary Research Center on Antimicrobial Resistance (MULTI-REZ), “Victor Babes” University of Medicine and Pharmacy, Eftimie Murgu Square, No. 2, 300041 Timisoara, Romania

**Keywords:** hand hygiene, healthcare workers, infection control, adenosine triphosphate, bioluminescence, healthcare-associated infections, ATP bioluminescence

## Abstract

Background and Objectives: Hand hygiene remains one of the most important measures for preventing healthcare-associated infections, yet its implementation in routine clinical practice remains suboptimal. This study aimed to evaluate whether repeated hand hygiene retraining combined with adenosine triphosphate (ATP) bioluminescence feedback was associated with reduced ATP-assessed residual organic contamination on the hands of healthcare workers in a real-world hospital setting. Materials and Methods: We conducted a single-center before-and-after interventional study in 2025 at the County Emergency Hospital of Deva, Romania. Hand contamination was assessed using ATP bioluminescence testing, and results were categorized as low residual contamination (≤20 RLU), intermediate residual contamination (21–59 RLU), or high residual contamination (≥60 RLU). One ATP assessment was performed before and one after a repeated hand hygiene retraining program delivered during 2025. The primary outcomes were the change in mean ATP values and the change in ATP-defined contamination categories between baseline and post-intervention assessment. Results: Mean ATP values decreased from 842 ± 210 RLU before the intervention to 312 ± 118 RLU after the intervention, corresponding to a 62.9% relative reduction (*p* < 0.01). At baseline, 17.1% of participants were classified as having low residual contamination, 25.7% as intermediate residual contamination, and 57.1% as high residual contamination. After the intervention, these proportions changed to 62.9%, 22.9%, and 14.3%, respectively (*p* < 0.01). Improvement was observed across all professional categories. Physicians showed the highest post-intervention proportion of compliant results (85.0%), followed by nurses (61.1%) and auxiliary staff (35.7%). ATP values decreased significantly in all seven departments, with relative reductions ranging from 61.0% to 63.4%. Conclusions: Repeated hand hygiene retraining combined with ATP bioluminescence feedback was associated with a substantial reduction in ATP-assessed residual hand contamination among healthcare workers in this hospital setting. The intervention was followed by both a marked reduction in ATP values and a favorable shift in ATP-defined contamination categories across departments and professional groups. Because ATP is an indirect and device-dependent marker of residual organic material, these findings should be interpreted cautiously and primarily as evidence of improved ATP-assessed hand cleanliness within the study setting rather than as direct evidence of WHO-defined hand hygiene compliance or microbiological decontamination.

## 1. Introduction

Healthcare-associated infections (HAIs) remain one of the most frequent and consequential complications of modern hospital care. In acute care hospitals, HAIs are associated with substantial morbidity, mortality, prolonged hospitalization, and increased healthcare costs [1,2,3]. Large prevalence studies from the United States and Europe have shown that a considerable proportion of hospitalized patients experience at least one HAI during admission, underlining the persistent burden of preventable infection in routine clinical practice [1,2]. This burden is further amplified by the growing prevalence of antimicrobial resistance and by the high resource use associated with surveillance, treatment, and prolonged inpatient care [2,3].

Among the available infection prevention and control measures, hand hygiene remains a cornerstone of HAI prevention because the hands of healthcare workers are the main vehicle for the transmission of pathogens between patients and within the healthcare environment [4,5]. For this reason, major guidance documents have consistently identified hand hygiene as one of the most important and universally applicable preventive strategies in healthcare [4,5]. Despite this strong consensus, however, implementation in real-world clinical settings remains challenging, and adherence to recommended hand hygiene practices is often lower than expected [5,6].

A major difficulty in this field is that the existing literature is highly heterogeneous. Systematic reviews have shown that hand hygiene studies vary considerably with respect to intervention type, targeted personnel, duration of follow-up, care setting, and reported outcomes [6,7,8,9,10]. Some studies focus primarily on compliance, whereas others assess broader infection prevention programs in which hand hygiene is only one component [7,8,9,10]. In addition, many interventions achieve only modest or short-term gains, and sustainability remains a recurring concern [7,8,10]. As a result, the evidence base is large but methodologically uneven, which complicates interpretation and limits direct translation into practice.

Another important limitation is that most hand hygiene studies measure whether hand hygiene occurred, rather than how well it was performed. Conventional surveillance methods such as direct observation remain widely used, but they are labor-intensive and prone to several well-recognized limitations, including observer effects and incomplete capture of actual practice [11]. Reviews of electronic and technology-assisted monitoring systems suggest that objective feedback and automated surveillance may improve hand hygiene performance, but the evidence remains heterogeneous and is still focused predominantly on compliance or event frequency rather than on the objective quality of the hand hygiene action itself [12,13]. Thus, although the need for better hand hygiene monitoring is widely recognized, there is still limited evidence on the value of rapid, objective, bedside assessment tools that can be used not only for measurement, but also for immediate feedback and staff retraining. One such approach is adenosine triphosphate (ATP) bioluminescence testing, which provides an indirect estimate of residual organic material on the hands but does not directly measure WHO-defined hand hygiene compliance or microbiological decontamination. At the same time, ATP bioluminescence should be interpreted cautiously, because threshold values are not universally standardized for hand assessment and results are influenced by device type, protocol, and sampling technique.

This study was undertaken to address a more specific gap in the literature: while many hand hygiene studies focus on whether hand hygiene was performed, fewer studies evaluate ATP-assessed residual contamination after hand hygiene in routine clinical practice, and there remains limited evidence on how such objective feedback tools may function as part of repeated retraining in real-world hospital settings. We aimed to evaluate whether ATP-guided feedback, combined with repeated retraining, was associated with reduced ATP-assessed residual organic contamination on the hands of healthcare workers in a real-world hospital setting. Specifically, we assessed changes in ATP values and in ATP-defined contamination categories between baseline and post-intervention measurements. In addition, we explored differences in ATP-defined contamination patterns across professional categories and hospital departments. By focusing on ATP-assessed residual contamination after hand hygiene, rather than on conventional observation alone, this study sought to provide a practice-oriented evaluation of a feedback-driven strategy that may be integrated into routine inpatient care.

## 2. Materials and Methods

### 2.1. Study Design and Participants

This study was designed as a single-center before-and-after interventional study evaluating the association of repeated hand hygiene retraining combined with ATP bioluminescence feedback with ATP-assessed residual hand contamination among healthcare workers. The study was conducted in 2025, from January until December, at the County Emergency Hospital of Deva, Romania, a county-level hospital serving the population of Hunedoara County.

The study consisted of a baseline pre-intervention ATP assessment and a post-intervention ATP reassessment after completion of the retraining program. Baseline ATP measurements were performed in January 2025, before the training program, and follow-up ATP measurements were performed in December 2025, after implementation of the educational intervention.

The analysis was restricted to the hand hygiene intervention component performed in seven hospital departments: intensive care, hematology, cardiology, pulmonology, gastroenterology, orthopedics, and urology. These departments were selected to reflect different clinical profiles, different levels of healthcare-associated infection risk, and a high degree of direct interaction between healthcare workers and patients.

Inclusion criteria for medical staff were: (1) active employment in one of the selected departments; (2) direct involvement in patient care activities; and (3) willingness to participate and provide written informed consent.

Participation in the study was voluntary. Thus, the study population consisted of all eligible healthcare workers who consented to participate. Of the 76 eligible healthcare workers, 70 agreed to participate and 6 declined; no random sampling was performed.

### 2.2. Educational Intervention

The intervention consisted of a periodic retraining program on hand hygiene, developed in accordance with World Health Organization recommendations. Training sessions were conducted in February, June, August, and December 2025. These four sessions were repeated retraining sessions with the same core educational content, rather than sequential modules with different material. The purpose of the repeated sessions was reinforcement of the same key hand hygiene principles over time. The educational content included: (1) the importance of hand hygiene in the prevention of healthcare-associated infections; (2) the WHO “Five Moments for Hand Hygiene”; (3) correct handwashing and hand disinfection technique; and (4) proper use of alcohol-based hand rubs.

All participants included in the study were required to attend all four retraining sessions as part of the study protocol, and all 70 enrolled participants completed the training program. ATP bioluminescence testing was incorporated into the study as an objective method for assessing residual organic contamination on the hands after hand hygiene. ATP measurements were obtained at two predefined time points only: once at baseline, before the educational intervention, and once after completion of the educational program. Thus, the four training sessions represented repeated reinforcement of the intervention during the year, whereas ATP-based outcome assessment was performed only at the pre-intervention and post-intervention time points.

### 2.3. ATP Bioluminescence Assessment of Hand Hygiene

Residual hand contamination after hand hygiene was assessed using ATP bioluminescence testing. ATP was used as an indirect marker of residual biological material, as it is present in microbial cells and human biological material. The bioluminescence method is based on the reaction between ATP and luciferase, producing light whose intensity is proportional to the amount of ATP present on the tested surface. Results were expressed as relative light units (RLU). Higher RLU values indicate a greater amount of residual organic material on the hands and, therefore, lower hand hygiene quality as assessed by ATP bioluminescence. ATP measurements were performed using the Hygiena EnSURE Touch luminometer (Hygiena LLC, Camarillo, CA, USA) together with UltraSnap surface ATP test devices, in accordance with the manufacturer’s instructions and the institutional testing protocol. For each scheduled assessment, samples were collected under standardized conditions from the palmar, thenar, interdigital, and dorsal areas of the hand, using uniform pressure and contact time. Because the hand represents an irregular sampling surface, a consistent swabbing technique was used for all participants, with the swab passed in a crisscross pattern while being rotated during sample collection. After sampling, the swab was returned to the device tube, the UltraSnap device was activated by breaking the Snap-Valve and squeezing the bulb twice to release the reagent, and the device was shaken for 5–10 s. The activated test device was then inserted into the EnSURE Touch luminometer in the upright position, the lid was closed, and measurement was initiated using the Run Test function. In accordance with the manufacturer’s instructions, each activated sample was read within 30 s, and results were displayed after approximately 10 s. One ATP determination was recorded at baseline and one after completion of the educational program for each participant. At each assessment time point, sampling was performed after completion of the participant’s hand hygiene procedure under standardized collection conditions.

Because ATP bioluminescence is not standardized across devices or protocols for hand assessment, the thresholds used in this study were treated as operational categories for within-study evaluation and feedback rather than as universally validated cutoffs. Their use was pragmatic and device-specific, based on the institutional protocol applied consistently with the EnSURE Touch/UltraSnap system throughout the study. Accordingly, ATP results were categorized as follows.

- ≤20 RLU: compliant;
- 21–59 RLU: partially compliant;
- ≥60 RLU: non-compliant.

In particular, the threshold of ≤20 RLU was used as the operational definition of low residual contamination within this study because it was prespecified in the institutional testing protocol. This cutoff should therefore be interpreted as a device-specific working threshold for classification and feedback within the present study, rather than as a universally accepted standard for hand-hygiene assessment.

### 2.4. Outcomes

The prespecified outcomes for this manuscript focused on ATP-assessed residual hand contamination measured by ATP bioluminescence. The primary outcomes were: (1) the change in mean ATP values (RLU) between baseline and post-intervention assessment; and (2) the change in the distribution of ATP-defined contamination categories between the two assessment points. The secondary outcomes were: (1) differences in ATP-defined contamination categories across professional groups at baseline and after the intervention; and (2) department-level changes in mean ATP values between the pre- and post-intervention assessments, including the relative reduction in ATP values.

### 2.5. Data Collection

Baseline ATP measurements were obtained in January 2025 before implementation of the educational intervention. The retraining program was then delivered periodically during the year, with repeated sessions in February, June, August, and December 2025. Follow-up ATP measurements were performed in December 2025 after completion of the educational program. For each participant, one pre-intervention and one post-intervention ATP determination were recorded, yielding 140 total measurements. Professional group and department data were collected for descriptive and subgroup analyses. Participant flow was also documented, including the number of eligible healthcare workers, the number who agreed to participate, and the number who declined participation.

### 2.6. Statistical Analysis

Statistical analysis included both descriptive and inferential methods. Continuous variables were summarized as means and standard deviations; where available, minimum and maximum values are also reported. Categorical variables were presented as absolute frequencies and percentages.

The change in mean ATP values before and after the intervention was evaluated using the paired-samples *t*-test. In addition, pre–post differences in mean ATP values were assessed separately within each department using paired-samples *t* tests, and the relative reduction in ATP values was calculated descriptively for each department. Changes in ATP-defined contamination categories between the pre- and post-intervention assessments were evaluated using the McNemar test for paired categorical data. Differences in compliance distribution between professional categories were analyzed using the chi-square test. Given the exploratory design and modest subgroup sizes, inferential findings were interpreted conservatively.

The level of statistical significance was set at *p* < 0.05. All statistical analyses were performed using SPSS version 26 (IBM Corp., Armonk, NY, USA).

### 2.7. Ethical Considerations

The study was approved by the Ethics Committee of the Emergency County Hospital of Deva (approval no. 1045 from 15 January 2025) and was conducted in accordance with the principles of the Declaration of Helsinki. Written informed consent was obtained from all participants before inclusion in the study. Participation was voluntary, and data were analyzed in anonymized form.

## 3. Results

### 3.1. Study Population

Across the seven selected hospital departments, 76 healthcare workers met the eligibility criteria and were invited to participate. Of these, 70 agreed to participate and 6 declined, yielding a participation rate of 92.1%. No random sampling was performed; the study population therefore consisted of all eligible healthcare workers who consented to participate. The final study population comprised 70 healthcare workers from seven hospital departments, generating 140 paired ATP measurements, with one assessment performed before the educational intervention and one after the intervention. The study population included 20 physicians (28.6%), 36 nurses (51.4%), and 14 auxiliary staff members (20.0%). Participants were recruited from the following departments: intensive care, hematology, cardiology, pulmonology, gastroenterology, orthopedics, and urology. The largest proportion of participants was represented by nurses, reflecting their frequent direct contact with patients and the hospital environment. Department-level distribution of the study population is shown in Table 1.

### 3.2. Baseline ATP-Defined Residual Contamination Categories

At baseline, ATP bioluminescence values indicated substantial residual organic contamination on the hands of the evaluated healthcare workers. Based on the predefined ATP categories used in this study, 12 of the 70 participants (17.1%) were classified as having low residual contamination, 18 (25.7%) as intermediate residual contamination, and 40 (57.1%) as high residual contamination. The overall baseline distribution is presented in Table 2.

Baseline ATP category distribution also differed significantly across professional categories. Physicians showed the highest proportion of low-residual-contamination results, whereas nurses and auxiliary staff had higher proportions of high-residual-contamination results. Specifically, 30.0% of physicians were in the low-residual-contamination category at baseline, compared with 13.9% of nurses and 7.1% of auxiliary staff. The detailed distribution is shown in Table 3. These differences were statistically significant (χ^2^ = 8.92, *p* = 0.012).

### 3.3. Change in ATP Values After Intervention

The educational intervention was associated with a marked reduction in ATP values. Mean ATP values decreased from 842 ± 210 RLU before training to 312 ± 118 RLU after training, corresponding to a mean relative reduction of 62.9%. Paired-sample *t*-test analysis demonstrated that this reduction was highly statistically significant (t = 18.4, *p* < 0.0001). This decrease reflected lower ATP-assessed residual organic contamination at the post-intervention assessment. The overall pre–post change is summarized in Table 4.

A consistent reduction in ATP values was observed across all seven departments. Department-specific relative reductions ranged from 61.0% to 63.4%, and all within-department comparisons were statistically significant (all *p* < 0.001). Department-level ATP changes are presented in Table 5.

### 3.4. Change in ATP-Defined Residual Contamination Categories After the Educational Intervention

The educational intervention was also associated with a substantial shift in ATP-defined contamination categories. After training, 44 of 70 participants (62.9%) were classified as having low residual contamination, 16 (22.9%) as intermediate residual contamination, and 10 (14.3%) as high residual contamination. Compared with baseline, the proportion of participants in the low-residual-contamination category increased from 17.1% to 62.9%, while the proportion in the high-residual-contamination category decreased from 57.1% to 14.3%. Paired categorical analysis using the McNemar test showed a statistically significant change in category distribution after training and ATP feedback (*p* < 0.0001). The overall shift in ATP-defined categories is summarized in Table 6.

### 3.5. Professional Category Analyses

Improvement in ATP-defined category distribution was observed across all professional categories. Among physicians, the proportion of low-residual-contamination results increased from 30.0% before intervention to 85.0% after intervention. Among nurses, this proportion increased from 13.9% to 61.1%, while among auxiliary staff it increased from 7.1% to 35.7%. Although post-intervention differences between professional groups remained statistically significant, they were attenuated compared with baseline (χ^2^ = 6.11, *p* = 0.047). Because several subgroup cells were small and the result was borderline, this finding should be interpreted cautiously. The post-intervention distribution by professional category is shown in Table 7.

## 4. Discussion

Our study showed that a repeated hand hygiene retraining program, combined with ATP bioluminescence feedback, was associated with a marked reduction in ATP-assessed residual hand contamination among healthcare workers. Mean ATP values decreased substantially after the intervention, and the distribution of ATP-defined categories shifted toward more favorable results, with a marked reduction in high-residual-contamination findings. Improvement was observed across all seven departments and all professional categories. Overall, these findings are consistent with the broader literature indicating that hand hygiene improvement is more successful when supported by structured, multimodal strategies rather than isolated educational messages alone [14,15].

The main finding of the study was the reduction in ATP values after the intervention. Because ATP bioluminescence reflects residual organic material rather than direct microbiological burden or WHO-defined hand hygiene compliance, the most appropriate interpretation is that residual hand contamination after hand hygiene was lower following repeated retraining. This interpretation is concordant with previous interventional studies in which multimodal strategies improved hand hygiene-related performance in real clinical settings. In the global quasi-experimental implementation of the WHO multimodal strategy, overall compliance improved substantially across sites [16]. Shen et al. reported improvements in both compliance and correctness after a WHO-based intervention [17], Randle et al. observed sustained gains after an educational intervention [18], and Baccolini et al. reported significant and persistent improvement following a multimodal ICU intervention [19]. Our findings are therefore aligned with the direction of previous research, although they should not be numerically equated with direct-observation studies because our outcome was ATP-assessed residual contamination rather than opportunity-based compliance.

At the same time, absolute RLU values should be interpreted cautiously. ATP bioluminescence results depend on the device used, the sampling technique, the hand areas sampled, the timing of sampling, and the thresholds adopted by the local protocol. For this reason, absolute RLU values are not readily interchangeable across studies, and no universally accepted ATP cutoff for hand assessment currently exists. In the present study, the value of ATP monitoring was therefore not to propose a universal threshold, but to apply the same device, protocol, and predefined categories consistently before and after the intervention within the same hospital setting.

An important strength of the study is its focus on an objective dimension of hand assessment that is not fully captured by routine observation alone. Wang et al. showed that most electronic hand hygiene monitoring studies evaluated compliance, whereas only a minority assessed technical quality, and standardized quality metrics remain lacking [20]. Szilágyi et al. demonstrated that even immediately after training, only 72% of staff achieved satisfactory hand coverage [21], while Stewardson et al. showed that immediate objective feedback improved the number of correctly performed poses during handrubbing [22]. Together with the broader review evidence summarized by Zhang et al., these findings support the view that objective assessment tools may provide complementary information rather than replace standard surveillance [13].

Not all studies of objective monitoring or feedback have reported uniformly positive effects. Aghdassi et al. found no significant overall improvement in hand hygiene compliance across intervention wards, although compliance before aseptic procedures increased significantly [23]. Iversen et al. likewise found no significant effect of data-driven feedback in two automatically monitored hospital departments and suggested that contextual factors such as time pressure, workplace culture, and leadership support may have influenced the result [24]. This heterogeneity is important when interpreting our findings. One possible explanation for the clearer improvement observed in our study is that the intervention was implemented in a single hospital across a defined set of departments and reinforced repeatedly during the year.

The differences observed between professional groups are also noteworthy. Physicians had the highest proportion of favorable ATP-defined results both at baseline and after the intervention, whereas nurses and especially auxiliary staff showed less favorable ATP-assessed results despite clear improvement. Similar patterns have been reported in some studies [17], although the literature is not uniform, as Ben Fredj et al. and Baccolini et al. also described variability across job categories [19,25]. This suggests that differences between professional groups are likely to be context-dependent and influenced by training background, task profile, workflow interruptions, and organizational factors. Qualitative data from Alshagrawi et al. support this interpretation [26]. In practical terms, repeated general retraining may improve ATP-assessed results across staff categories, but targeted strategies may still be needed for groups at higher risk of unfavorable results. For auxiliary staff in particular, future implementation could include shorter hands-on sessions, role-specific demonstrations, direct supervised practice, simpler visual reminders at the point of care, and more frequent immediate feedback. These measures may be especially relevant for staff whose workflow is highly task-driven and less likely to benefit fully from general didactic retraining alone. These between-group findings should nevertheless be interpreted cautiously because subgroup counts were modest and the post-intervention comparison was only borderline significant.

Another relevant finding was the consistency of ATP reduction across all included departments. This suggests that the intervention effect was not confined to a single ward type, but may be applicable across services with different clinical profiles. This is practically important because implementation studies often show variability between departments. Elia et al. reported improvement in all wards included in their nudge-based intervention [27], whereas Ben Fredj et al. found gains in some departments but not uniformly across all settings [25]. The more consistent pattern observed in our study may reflect the standardized institutional delivery of retraining across departments. Fukada et al. likewise described ATP bioluminescence as an easy-to-perform, on-the-spot, objective method, supporting its potential usefulness as an educational or audit tool in routine hospital practice [28]. In addition, no other hospital-wide infection-control initiatives specifically targeting hand hygiene were introduced at County Emergency Hospital of Deva during 2025, which reduces concern that the observed pre–post changes were driven mainly by parallel institutional interventions.

The sample size should also be interpreted in the context of the study design. This was a pragmatic single-center intervention conducted in seven departments, and the study population represented 70 of 76 eligible healthcare workers in those departments. Thus, although the absolute sample size was modest, participation coverage among eligible staff was high. At the same time, because participation was voluntary and 6 eligible workers declined participation, some selection bias cannot be excluded, and the findings may overestimate improvement if those who agreed to participate were more motivated or more receptive to training than those who declined.

Because the study used only two fixed measurement points, the durability of the observed improvement remains uncertain. In practical terms, maintenance of these gains would likely require continued periodic retraining, repeated ATP-based feedback at scheduled intervals, integration of hand assessment into routine institutional audit, and targeted refresher sessions for units or staff groups with less favorable results. This interpretation is also consistent with the broader project recommendations, which support continued monitoring, audit integration, and differentiated educational follow-up.

Several limitations should be acknowledged. First, this was a single-center pre–post study with a relatively small sample, and no parallel control group was included. Therefore, causal inference should be made cautiously, because secular changes, institutional emphasis on infection prevention, product availability, or other unmeasured contextual factors may have contributed to the observed improvement. Second, participation was voluntary among eligible staff. Although 70 of 76 eligible healthcare workers participated, the possibility of selection bias remains because staff who agreed to participate may have been more cooperative, more motivated, or more receptive to hand hygiene training than those who declined. Third, measurements were obtained at only two fixed time points, which limits the ability to assess the trajectory and long-term sustainability of the effect throughout the year. Fourth, although ATP bioluminescence provides rapid and objective data, it remains an indirect marker of residual organic contamination and does not directly quantify microbial burden, transmission risk, or clinical outcomes. ATP methodology is also not standardized across devices, and threshold values may differ substantially between instruments and protocols. In addition, ATP readings can be influenced by disinfectant chemistry and may show limited sensitivity at low levels of contamination. For these reasons, our findings should be interpreted as evidence of improved ATP-assessed hand hygiene quality rather than proof of reduced infection risk or direct microbiological decontamination.

We also did not evaluate microbiological cultures or healthcare-associated infection outcomes within the scope of this manuscript, so the downstream clinical relevance of the observed ATP reduction remains unknown. Even so, the study has several strengths, including its real-world setting, inclusion of multiple departments and professional groups, standardized retraining during the study period, and use of an objective pre–post assessment approach. Future research should build on these findings through multicenter controlled studies with larger samples, repeated interim assessments, and parallel measurement of direct-observation compliance, ATP results, and microbiological endpoints. It would also be valuable to investigate whether tailored interventions for different professional groups, particularly nurses and auxiliary staff, yield more equitable improvement, and whether validated device-specific ATP thresholds for hand assessment can be developed. More rigorous studies are also needed to determine whether objective improvements in hand hygiene quality translate into measurable reductions in healthcare-associated infections over time.

## 5. Conclusions

In conclusion, repeated hand hygiene retraining combined with ATP bioluminescence feedback was associated with reduced ATP-assessed residual hand contamination among healthcare workers in this hospital setting. The intervention was followed by a marked reduction in ATP values and by a favorable shift in ATP-defined contamination categories across departments and professional groups. These findings suggest that objective feedback-based strategies may have practical value as complementary tools for hand hygiene education and audit in routine inpatient care. At the same time, ATP should be interpreted as an indirect and device-dependent marker of residual organic contamination rather than a direct measure of WHO-defined hand hygiene compliance, microbiological decontamination, or clinical risk reduction. Because this was a single-center before-and-after study with voluntary participation and no control group, the findings should be interpreted cautiously. Further controlled multicenter studies are needed to assess long-term sustainability and to determine whether improvements in ATP-assessed outcomes translate into better infection prevention outcomes.

## Figures and Tables

**Table 1:** Distribution of study participants by department and professional category.

Department	Physicians, n	Nurses, n	Auxiliary Staff, n	Total, n
Intensive care	4	6	2	12
Hematology	2	4	2	8
Cardiology	3	5	2	10
Pulmonology	3	5	2	10
Gastroenterology	2	4	2	8
Orthopedics	3	7	2	12
Urology	3	5	2	10
Total	20	36	14	70

**Table 2:** Baseline ATP-based Residual Contamination Categories (pre-intervention).

ATP Category	n	%
Low residual contamination (≤20 RLU)	12	17.1
Intermediate residual contamination (21–59 RLU)	18	25.7
High residual contamination (≥60 RLU)	40	57.1
Total	70	100.0

**Table 3:** Baseline ATP-based residual contamination categories by professional category.

Professional Category	Low Residual Contamination, n (%)	Intermediate Residual Contamination, n (%)	High Residual Contamination, n (%)
Physicians (n = 20)	6 (30.0)	7 (35.0)	7 (35.0)
Nurses (n = 36)	5 (13.9)	8 (22.2)	23 (63.9)
Auxiliary staff (n = 14)	1 (7.1)	3 (21.4)	10 (71.4)

**Table 4:** ATP values before and after intervention.

Time Point	Mean RLU	SD (Min–Max)
Pre-intervention	842	210 (390–1351)
Post-intervention	312	118 (140–533)

**Table 5:** Department-level ATP values before and after intervention.

Department	Pre-Intervention RLU ± SD (Min–Max)	Post-Intervention RLU ± SD (Min–Max)	Reduction, %	*p* Value
Intensive care	910 ± 225 (460–1351)	355 ± 89 (177–533)	61.0	<0.001
Hematology	870 ± 214 (445–1308)	330 ± 83 (164–496)	62.1	<0.001
Cardiology	780 ± 191 (390–1171)	290 ± 73 (144–436)	62.8	<0.001
Pulmonology	820 ± 205 (426–1132)	305 ± 76 (153–457)	62.8	<0.001
Gastroenterology	760 ± 199 (392–1088)	280 ± 70 (140–420)	63.2	<0.001
Orthopedics	890 ± 238 (454–1335)	340 ± 85 (170–510)	61.8	<0.001
Urology	860 ± 210 (439–1306)	315 ± 79 (157–473)	63.4	<0.001

**Table 6:** ATP-based residual contamination categories before and after intervention.

Time Point	Low Residual Contamination, n (%)	Intermediate Residual Contamination, n (%)	High Residual Contamination, n (%)
Pre-intervention	12 (17.1)	18 (25.7)	40 (57.1)
Post-intervention	44 (62.9)	16 (22.9)	10 (14.3)

**Table 7:** Post-intervention ATP-defined residual contamination categories by professional category.

Professional Category	Low Residual Contamination, n (%)	Intermediate Residual Contamination, n (%)	High Residual Contamination, n (%)
Physicians (n = 20)	17 (85.0)	2 (10.0)	1 (5.0)
Nurses (n = 36)	22 (61.1)	10 (27.8)	4 (11.1)
Auxiliary staff (n = 14)	5 (35.7)	4 (28.6)	5 (35.7)

## Data Availability

The data presented in this study are available on request from the corresponding author due to privacy and ethical restrictions related to the small number of healthcare workers included in each department and professional category, which may create a potential risk of re-identification even after anonymization. Data access may be granted upon reasonable request and, where applicable, with permission from the Ethics Committee of the Emergency County Hospital of Deva.

## Abbreviations

The following abbreviations are used in this manuscript:

HAI	Healthcare-Associated Infection
ATP	Adenosine Triphosphate
RLU	Relative Light Unit
WHO	World Health Organization
ICU	Intensive Care Unit
SD	Standard Deviation
